# Fusion of Renewable Ring Resonator Lasers and Ultrafast Laser Inscribed Photonic Waveguides

**DOI:** 10.1038/srep32668

**Published:** 2016-09-07

**Authors:** Hengky Chandrahalim, Stephen C. Rand, Xudong Fan

**Affiliations:** 1 Department of Biomedical Engineering, University of Michigan, 1101 Beal Ave., Ann Arbor, MI 48109, USA; 2 Department of Electrical Engineering and Computer Science, University of Michigan, 1301 Beal Ave., Ann Arbor, MI 48109, USA

## Abstract

We demonstrated the monolithic integration of reusable and wavelength reconfigurable ring resonator lasers and waveguides of arbitrary shapes to out-couple and guide laser emission on the same fused-silica chip. The ring resonator hosts were patterned by a single-mask standard lithography, whereas the waveguides were inscribed in the proximity of the ring resonator by using 3-dimensional femtosecond laser inscription technology. Reusability of the integrated ring resonator – waveguide system was examined by depositing, removing, and re-depositing dye-doped SU-8 solid polymer, SU-8 liquid polymer, and liquid solvent (toluene). The wavelength reconfigurability was validated by employing Rhodamine 6G (R6G) and 3,3′-Diethyloxacarbocyanine iodide (CY3) as exemplary gain media. In all above cases, the waveguide was able to couple out and guide the laser emission. This work opens a door to reconfigurable active and passive photonic devices for on-chip coherent light sources, optical signal processing, and the investigation of new optical phenomena.

On-chip micro-lasers with the dye gain medium either dissolved in solvent or doped in polymer have recently been under intensive research for integrated tunable coherent light sources with a large spectral coverage. Compared to other types of on-chip optical micro-cavities such as distributed feedback and Fabry–Pérot cavities, micro-ring resonators have advantages in relatively high quality (Q)-factors, simple geometries, small footprints, and the ability to accommodate a broad range of lasing emission wavelengths. Previously, micro-ring resonator based dye lasers built on soft materials such as polydimethylsiloxane were demonstrated^1^,^2^. However, the modest flexural rigidity and limited chemical compatibility impose challenges to operate those devices under practical conditions. Ring resonator lasers made of much more rigid solid-state materials have also been explored, which offer structural stability and integrity for laser operations^3^,^4^,^5^,^6^,^7^,^8^. Particularly, the large array production of dye-doped polymer microgoblet ring resonator lasers was achieved by a single-step of Deep-UV (DUV) lithography followed by thermal reflow. While this method shows great potential to manufacture solid-state micro-lasers economically^8^, it lacks of the accuracy to define the final shape and size of the ring resonators after the reflowing process.

Very recently, we have developed highly versatile ring resonator dye lasers built on fused-silica substrates^9^,^10^. In those micro-dye lasers, hollow ring-shaped structures were fabricated by using 3-dimensional (3-D) femtosecond laser nanofabrication technique^9^. Their fabrication is economical and based on standard photolithography^10^. These ring resonators were filled with a high-index dye solution or dye-doped solid polymer to form well-confined micro-ring cavity lasers. They offer a plethora of advantages over existing ring resonator dye lasers, including well-defined ring resonator geometries (shape and size), inherent mechanical and chemical robustness, and regeneration capability (*i.e*., dyes can be replaced in the solvent or polymer inside the hollow ring structures).

Despite the aforementioned advantages provided by the micro-ring resonator lasers, guiding and coupling out the laser emission in a reliable and flexible manner remain challenging. Oftentimes, the free-space coupling method is used to collect the radiated light from the proximity of the chip. While simple, this technique has several substantial drawbacks, such as the lack of directionality in laser emission, the absence of guided laser emission within the chip plane, and the collection of a large and undesired fluorescent background. Over the years, a few in-plane light guiding schemes, such as lateral and vertical couplings, have been explored. One possible lateral coupling method is to fabricate a solid fused-silica rib or ridge waveguide next to the ring resonator. However, the physical construction of the coupling region between the resonator and the waveguide using conventional lithography and etching processes is complicated and may involve expensive and time-consuming electron-beam (e-beam) lithography. Furthermore, the rib and ridge waveguides typically require an isolation layer underneath to prevent optical leakage to the substrate^11^,^12^,^13^,^14^. Another lateral coupling method uses a waveguide formed by filling a fluidic channel with high-index liquids to transport light from the adjacent ring resonator^1^,^2^,^15^,^16^,^17^. This type of waveguide can be conveniently fabricated together with the optical resonators in the same lithography and etching steps. Nevertheless, reliable liquid delivery to the cavity still calls for further investigations. Furthermore, the narrow coupling gap between the resonator and the waveguide is difficult to define. In most cases, the waveguide and the ring resonator are simply fluidically connected. In addition to lateral coupling, vertical coupling between the ring resonators and waveguides have been previously demonstrated^18^,^19^,^20^,^21^,^22^, in which the vertical coupling gap can be constructed very precisely by depositing a thin low-refractive-index material or sacrificial layer between the ring resonators and the waveguides. Although this technique promises to integrate various optical components with very controllable distances in vertical dimension, it involves slow, complicated, and strenuous fabrication processes^18^,^21^,^23^.

Here, we have demonstrated the monolithic integration of photolithographically fabricated ring resonators and optical waveguides inscribed by an ultrafast laser. The ring resonator is an open channel that can accommodate any solid and liquid gain media whose refractive indices are higher than that of fused-silica and the waveguide can out-couple and guide the laser emission from the ring resonator. The 3-D schematics of the gain media doped solid and liquid ring resonator lasers with embedded optical waveguides are illustrated in Fig. 1. Our uniquely developed hybrid fabrication process exploits a nonlinear light-matter interaction that locally induces a refractive index increase (core-cladding index difference of ~5.25 × 10^−3^)^24^ and is capable of creating arbitrary 3-D shapes to out-couple and direct the laser emission from the optical resonators in any direction within the fused-silica substrate. Unlike the aforementioned integrated ring resonator and waveguide systems^2^,^15^,^16^, the waveguides presented here are convenient and economical, and can be rapidly and accurately patterned with a single-step maskless ultrafast laser writing process at room temperature and pressure. They are well protected within the circumambient fused-silica and physically isolated from the laser gain media deposited in the ring cavity, thereby enabling a high performance laser system with high mechanical strength, durability, broad chemical compatibility, and excellent thermal stability. The renewability of the laser system was examined by depositing, removing, and re-depositing dye-doped solid polymers, liquid polymers, and solvents in the resonator. The wavelength reconfigurability was validated by employing Rhodamine 6G (R6G) and 3,3′-Diethyloxacarbocyanine iodide (CY3) as prototypical gain media. A lasing threshold of 315 nJ/mm^2^ was achieved with R6G-doped solid polymer, denoting a remarkably low threshold compared to any other solid-state dye-doped polymer laser fabricated with standard lithography process on a chip.

## Methods

**Figure 1:**
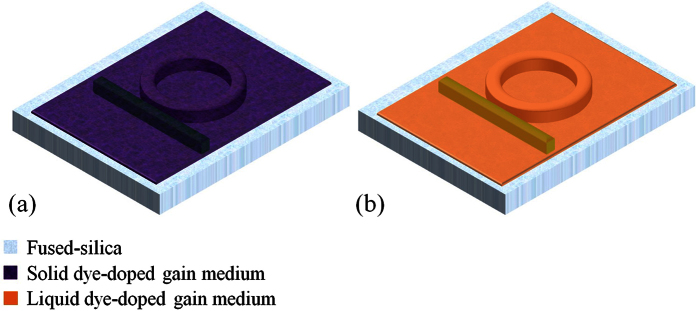
3-D schematics of the fused-silica hollow ring resonator coated with a gain medium doped high refractive index (**a**) solid polymer and (**b**) liquid. The optical waveguide inscribed by a femtosecond laser is embedded inside the fused-silica wafer, next to the ring cavity.

**Figure 2:**
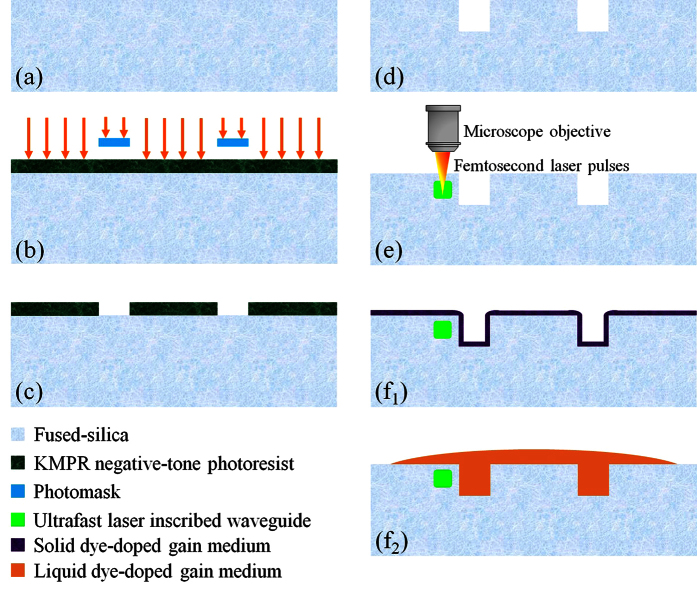
The fabrication process flow of the ring resonators: (**a**) 500 μm thick fused-silica wafer. (**b**) 16 μm thick KMPR negative-tone photoresist was spin-coated on the top of the fused-silica wafer and exposed to the UV light. (**c**) The developed photoresist on the fused-silica wafer. (**d**) The fused-silica wafer after a dry-etching process by using a reactive-ion etcher. (**e**) The optical waveguide was inscribed ~800 nm away from the ring resonator by using femtosecond laser pulses. (f_1_) 800 nm thick layer of gain medium doped polymer was deposited and cured on the surface of the cavity. (f_2_) A high refractive index liquid (liquid polymer or solvent) gain medium was deposited in the ring cavity.

The integrated dye lasers in this work were fabricated in fused-silica (the “host”) as ring-shaped resonators, as illustrated in Fig. 1. The dye was dissolved (or doped) in a high refractive index (>1.46) solvent (or polymer) and preferentially deposited within the ring cavity. The ring resonator host structure was designed to have inner and outer radii and depth of 110, 150, and 30 μm, respectively. We chose a relatively large ring diameter because we would like to eliminate the radiation loss issue for the proof of concept device demonstration. They were fabricated with a wafer level single-mask bulk micromachining process as outlined in Fig. 2. A 100 mm diameter fused-silica wafer was first cleaned in a Piranha solution (H_2_SO_4_ + H_2_O_2_) at 60 °C for 10 minutes. Next, the wafer was spin-coated with a 16 μm thick negative-tone photoresist (KMPR) and soft-baked at 100 °C for 6 minutes. A Suss MicroTec MA/BA-6 contact aligner was used to expose the wafer to UV light. The wafer was then baked at 100 °C for 3 minutes and developed with a TMAH (Tetramethylammonium hydroxide) based photoresist developer. A reactive ion glass etcher was used to etch the fused-silica wafer. Our fused-silica etching recipe has been characterized to consistently provide the side-wall roughness of better than 30 nm. The chamber pressure, coil power, and platen power were set at 4 mTorr, 1400 W, and 300 W, respectively. The gasses used in this etching process were C_4_F_8_ and He with flow rates of 10 and 174 sccm, respectively. After the etching process, the wafer was diced into 1 cm × 1 cm chips with an ADT 7100 dicing saw.

**Figure 3:**
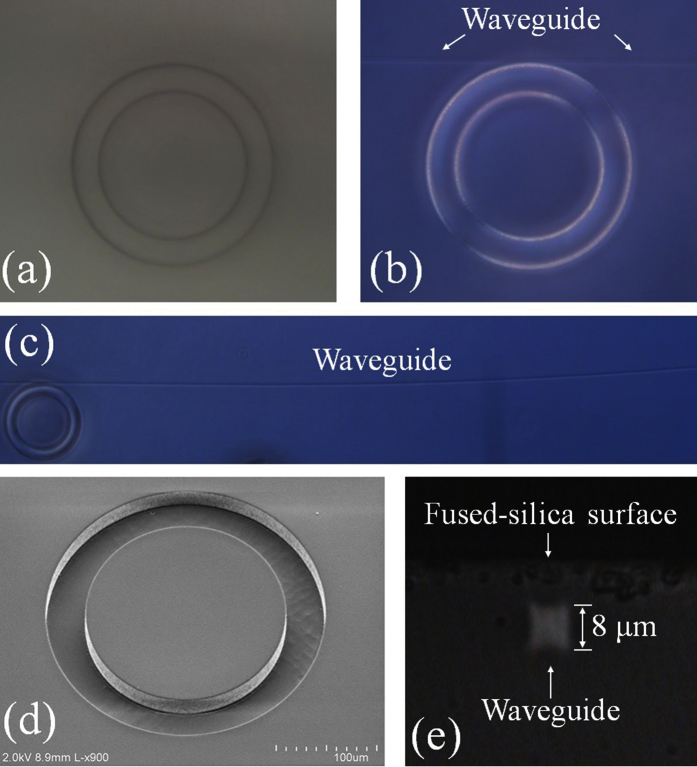
(**a**) A bright field microscope image of the fabricated ring resonator with inner and outer radii and depth of 110, 150, and 30 μm, respectively. (**b**) A differential interference contrast (DIC) microscope image of the same ring resonator revealing the refractive index modified optical waveguide inscribed by a femtosecond laser next to the resonator. (**c**) A DIC microscope image of the same ring resonator and waveguide displayed in a larger field of view. (**d**) A scanning electron microscope (SEM) image of the identical ring resonator exhibiting smooth and crack-free inner-wall of the ring cavity around the vicinity where the optical waveguide was inscribed by femtosecond laser pulses. (**e**) A cross-sectional view of the waveguide at the edge of the chip, demonstrating distinct guidance and confinement of the microscope illumination light.

The optical waveguide inscription process was performed with an ultrafast laser (wavelength λ = 800 nm, pulse width 100 fs, pulse energy 0.3 μJ, and repetition rate 250 kHz) focused with a 50× objective into the previously diced fused-silica chip, as shown in Fig. 2(e). The laser spot diameter on the sample was approximately 1 μm. Multiple-pass laser writing in 3-D was executed to form an optical waveguide with cross-sectional dimensions of 8 μm × 8 μm. The depth of the waveguide and the fused-silica gap between the waveguide and the ring resonator were designed to be 15 μm and 800 nm, respectively. The scanning speed of the substrate varied from 0.05 to 2 mm/s, depending on the local geometry of the ring cavities. The femtosecond laser’s pulse energy and repetition rate was very carefully optimized during the waveguide inscription to prevent crack formation along the inner wall of the ring cavity. Detailed waveguide writing parameters have been previously presented elsewhere^24^,^25^,^26^. The loss of the index modified optical waveguide in the same fused-silica substrate has been characterized to be consistently less than 0.15 dB/cm at 1550 nm. The bright field microscope image of the fabricated ring resonator host prior to the deposition of the gain medium is shown in Fig. 3(a). Since the photo-induced refractive index change is less than 1% of the nominal refractive index of fused-silica, the inscribed waveguide is not visible under bright field microscope imaging. However, it is evident under a differential interference contrast (DIC) microscope (Fig. 3(b,c)). The scanning electron microscope (SEM) image in Fig. 3(d) shows that the ring resonator host was crack-free. Guidance and confinement of the microscope illumination light from the cross-sectional perspective of the waveguide at the edge of the chip is presented in Fig. 3(e). Once the ring resonator host and waveguide are completely constructed, various gain media in liquids and polymers can easily be deposited onto and removed from the fused-silica chip (see Figs 1 and 2). Due to high mechanical and chemical robustness of the device, the above cycles can last indefinitely.

## Experimental

The objective of this work was to demonstrate a reusable and reconfigurable laser platform with on-chip directional laser output coupling. The reusable aspect of the device was investigated by a series of experiments involving the deposition, removal, and re-deposition of dye-doped solid polymer, liquid polymer, and solvent. The wavelength reconfigurability was demonstrated by replacing R6G with CY3 as the sample gain medium.

The solid-state ring resonators that support the whispering-gallery modes (WGMs) were filled with dye-doped polymer having a refractive index higher than that of the surrounding fused-silica, as illustrated in Fig. 2(f1). The dye was first dissolved in ethanol (10 mM dye concentration) and subsequently mixed with SU-8 in the liquid form at a ratio of 10% (ethanol + dye) to 90% (SU-8, refractive index ~1.6) in the ultrasonic bath for 60 minutes. The solution was then spin-coated onto the ring resonator host and baked for 30 minutes at 95 °C to evaporate the solvent and solidify the polymer. Since the solidified polymer was enclosed by a mechanically robust circumambient fused-silica, the conventional post-baking UV illumination for polymer cross-linking was unnecessary, allowing for easy removal of the polymer and re-use of the laser. In our current work, SU-8 was extracted with Remover PG solution (MicroChem) and rinsed with isopropanol (IPA). For liquid phase deposition, the dye was dissolved in liquid SU-8 or toluene (refractive index ~1.5) at a concentration of approximately 1 mM and then drop-coated onto the fused-silica chip, as shown in Fig. 2(f2). The removal of the active liquid gain medium required for re-use of the laser was again accomplished by rinsing the chip with Remover PG solution.

**Figure 4:**
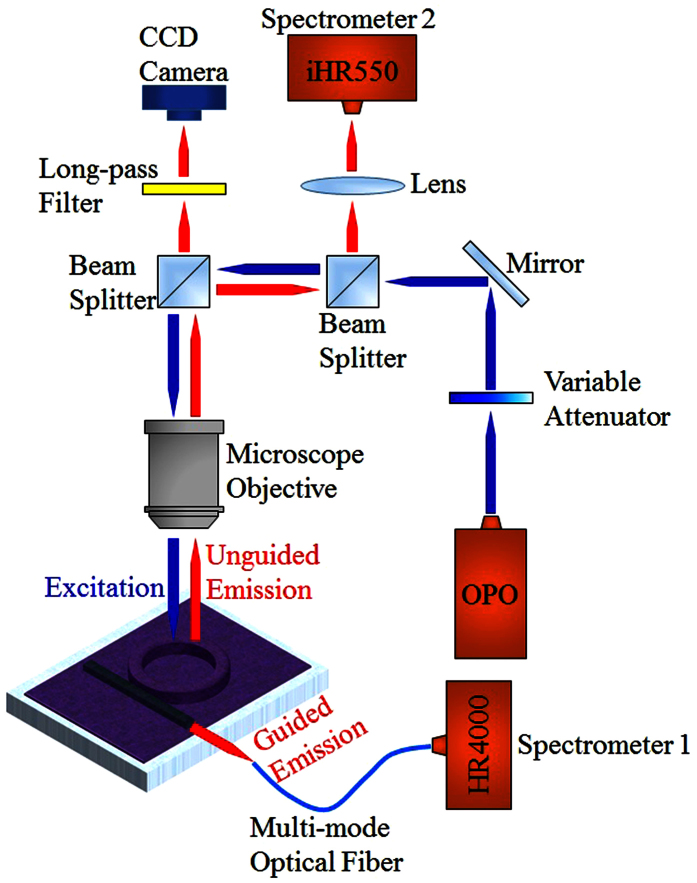
Schematic of the measurement setup.

The measurement setup is outlined in Fig. 4. Nanosecond pulses from an optical parametric oscillator (OPO) with a repetition rate of 20 Hz and pulse width of 5 ns were focused to a spot area of 0.03 mm^2^ on the device. Two independent detection paths were used to monitor light from the device. One path acquired guided laser output from the side of the chip and the other intercepted unguided laser light scattered out the top of the device. The optically guided output was collected by a multimode optical fiber connected to an Ocean Optics HR4000 spectrometer with a resolution of 0.7 nm (Spectrometer 1). Unguided emission from the top of the cavity was captured by a microscope objective and directed to a Horiba iHR550 spectrometer with a resolution of 0.2 nm (Spectrometer 2).

## Results

We first theoretically estimated the coupling coefficient (κ) between the ring resonator and ring resonator using the coupled-mode theory. The detailed calculation and the related results are presented in Figs S1 and S2, as well Table S1. The κ of our current device was approximately 0.02, which corresponds to a coupling Q-factor of approximately 2.2 × 10^7^.

**Figure 5:**
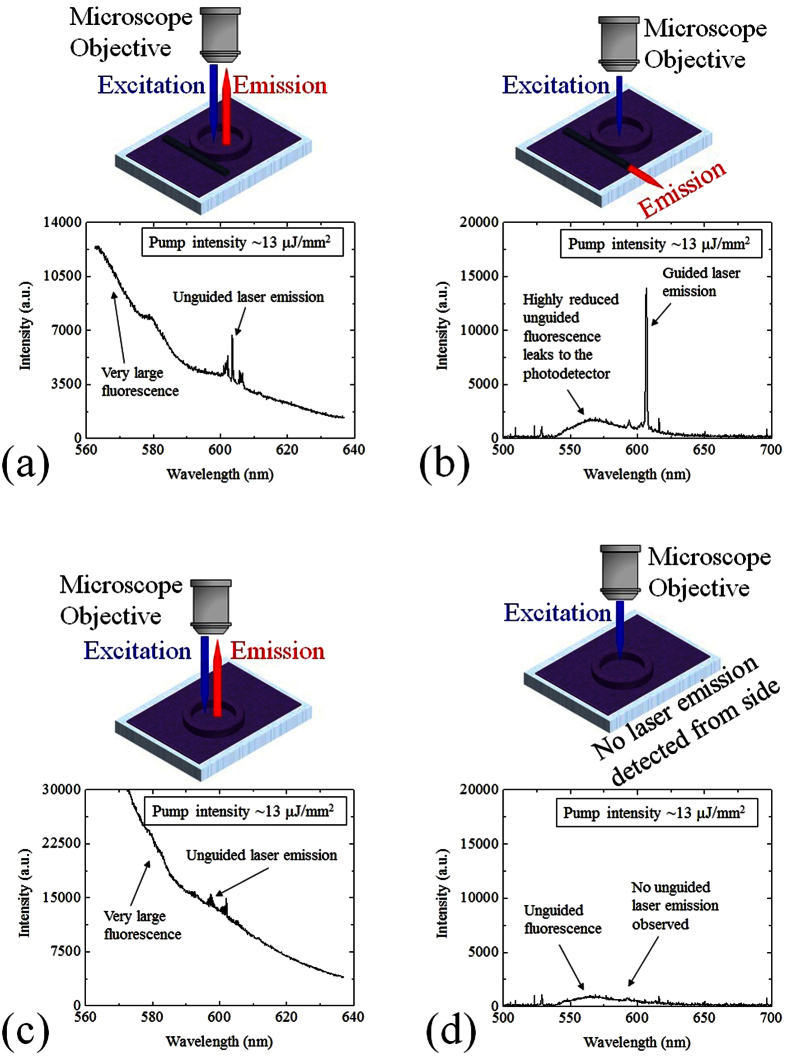
Series of measurements to validate the laser emission through the refractive index modified optical waveguide. (**a**) A free-space detected lasing spectrum of a solid R6G-doped SU-8 ring laser with an optical waveguide written next to it. A Horiba iHR550 (Spectrometer 2 in Fig. 4) with a 600 g/mm grating was used to record the multimode laser emission and fluorescent background emitted by the dye molecules. (**b**) A lasing spectrum from the same ring laser in (**a**) was detected simultaneously at the output port of the optical waveguide by Ocean Optics HR4000 spectrometer with 0.7 nm resolution (Spectrometer 1 in Fig. 4). (**c**) A free-space detected lasing spectrum of a solid R6G-doped SU-8 ring laser with no optical waveguide written next to it. A Horiba iHR550 spectrometer (Spectrometer 2 in Fig. 4) was used to record the multimode laser emission and fluorescent background emitted by the dye molecules. (**d**) No laser emission from the same ring resonator in (**c**) was detected from the side of the device. The pump intensity of 13 μJ/mm^2^ at 530 nm wavelength was applied to all measurements in (**a–d**).

We performed a series of experiments to examine the capability of the inscribed optical waveguide to couple out and guide the laser emission. Fig. 5(a) shows the laser emission spectrum monitored via the free-space coupling through the top of the cavity. Multi-mode lasing peaks are seen to be superimposed on a very intense fluorescent background from dye molecules outside the relevant mode volume that did not participate in laser action. In contrast, strong and distinct laser peaks were observed at the output port of the optical waveguide with significant suppression of the fluorescent background. Principally, only the laser emission from the WGM can be efficiently coupled out and guided by the side waveguide. Conventional fluorescence, due to the large phase mismatch, cannot be coupled out or guided. The small fluorescent hump around 560 nm in Fig. 5(b) originated from unguided fluorescence that leaked to the collection fiber, which had a core diameter of 400 μm. Figure S3 in the Supplementary Material shows that the lasing peaks diminished significantly when the collection fiber was translated slightly with respect to the waveguide output port.

In order to further verify that the laser emission collected from the side of the chip was not the unguided laser emission that leaked laterally through the fused-silica substrate, we examined the output of a second ring resonator that lacked the optical waveguide under identical conditions, collecting light through the side of the substrate. Again, laser emission along with intense fluorescent background was observed via the light emitted through the top of the device using free-space coupling. However, only weak fluorescence was detected using collection fiber at the edge of the chip. Our measurements consistently indicated that laser emission took place in the ring resonator and could be coupled out and guided via an adjacent waveguide.

**Figure 6:**
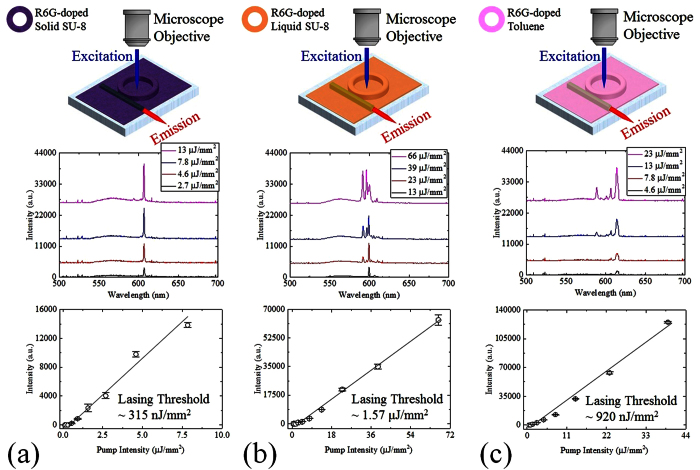
Lasing spectra of R6G-doped solid SU-8 (**a**), R6G-doped liquid SU-8 (**b**), and R6G-doped toluene (**c**) ring lasers were detected at the output port of the optical waveguide by Ocean Optics HR4000 spectrometer with 0.7 nm resolution (Spectrometer 1 in Fig. 4). The spot size of the OPO pumping laser beam was approximately 0.03 mm^2^. All spectra are vertically shifted for clarity. Spectrally integrated laser outputs (605–608 nm, 585–605 nm, and 585–618 nm for R6G-doped solid SU-8, R6G-doped liquid SU-8, and R6G-doped toluene ring resonator lasers, respectively) as a function of the pump intensity extracted from the corresponding laser spectra. The extracted lasing thresholds for the R6G-doped solid SU-8, R6G-doped liquid SU-8, and R6G-doped toluene ring resonator lasers were approximately 0.315, 1.57, and 0.92 μJ/mm^2^, respectively. Error bars were obtained with 3 measurements. The excitation wavelength for all measurements in (**a–c**) was 530 nm.

After validating the integrity of the laser-inscribed photonic waveguide, we comprehensively characterized a micro-ring resonator containing R6G-doped SU-8 in solid form. The ring laser was systematically excited with increasing pump intensity at 530 nm wavelength and the lasing spectra were monitored from the top and side of the chip as described earlier in Fig. 4. The spot size of the OPO pumping laser beam was approximately 0.03 mm^2^. The guided laser output centered around 605 nm was detected at the output port of the optical waveguide by an Ocean Optics HR4000 spectrometer (Spectrometer 1 in Fig. 4) as indicated in Fig. 6(a). Measurements of the laser emission (spectrally integrated over the range 605–608 nm) as a function of pump intensity were made to determine a single-pulse lasing threshold of ~315 nJ/mm^2^. Compared to the previously reported solid-state dye-doped polymer lasers fabricated with a standard lithography process on a chip, this is a remarkably low threshold. Operation of the laser was found to be multimode by analyzing the output with a Horiba iHR550 spectrometer (Spectrometer 2 in Fig. 4) which had a resolution of 0.2 nm. This is shown in Fig. S4(a).

One of the major benefits of our ring resonator platform over the other existing on-chip laser technologies is its capability for re-use, which is easily accomplished by removing and re-depositing different types and phases of gain media in a single device. For example, it was straightforward to remove the solid dye-doped polymer from the chip using Remover PG solution and subsequently deposit an R6G-doped SU-8 solution by drop-coating the cavity as illustrated in Figs 1(b) and 2(f2). Following this operation, we performed the customary characterization steps and obtained the results summarized in Fig. 6(b). The laser emission (in this case spectrally integrated over the range 585–605 nm) versus pump intensity indicated a lasing threshold of approximately 1.57 μJ/mm^2^.

Here we have demonstrated the operation of a dye-doped liquid polymer laser with a kinematic viscosity of ~2.5 cSt inside an integrated ring cavity platform. However, many on-chip optofluidic systems incorporate long microfluidic channels with very small cross-sectional dimensions. In less accessible devices of this type, it is essential to employ fluidic gain medium carriers with kinematic viscosity comparable to or less than water (for which the kinematic viscosity is ~1 cSt, at room temperature). We utilized toluene (refractive index ~1.5) which has a kinematic viscosity of ~0.68 cSt at room temperature as a model fluidic gain medium carrier to explore less viscous compounds. In this case, the dye-doped liquid polymer on the chip was removed so that an R6G-doped toluene solution (~1 mM) could be drop-coated on the cavity as illustrated in Figs 1(b) and 2(f2). Identical characterization procedures were then used to acquire the measurement results summarized in Fig. 6(c). The laser output (spectrally integrated over the range 585–618 nm) was again recorded as a function of the pump intensity and yielded a lasing threshold of approximately 920 nJ/mm^2^. This low lasing threshold compares very well with that of other optofluidic dye lasers fabricated with standard lithography on a chip.

**Figure 7:**
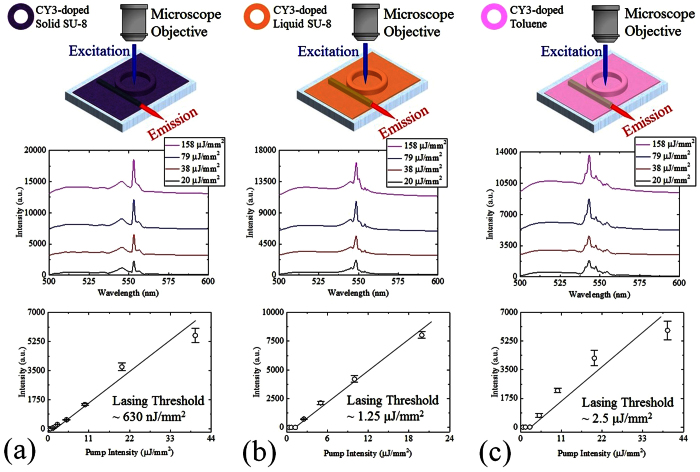
Lasing spectra of CY3-doped solid SU-8 (**a**), CY3-doped liquid SU-8 (**b**), and CY3-doped toluene (**c**) ring lasers were detected at the output port of the optical waveguide by Ocean Optics HR4000 spectrometer with 0.7 nm resolution (Spectrometer 1 in Fig. 4). The spot size of the OPO pumping laser beam was approximately 0.03 mm^2^. All spectra are vertically shifted for clarity. Spectrally integrated laser outputs (551–555 nm, 540–555 nm, and 540–545 nm for CY3-doped solid SU-8, CY3-doped liquid SU-8, and CY3-doped toluene ring resonator lasers, respectively) as a function of the pump intensity extracted from the corresponding laser spectra. The extracted lasing thresholds for the CY3-doped solid SU-8, CY3-doped liquid SU-8, and CY3-doped toluene ring resonator lasers were approximately 0.63, 1.25, and 2.5 μJ/mm^2^, respectively. Error bars were obtained with 3 measurements. The excitation wavelength for all measurements in (**a–c**) was 480 nm.

Thus we have successfully demonstrated laser operation with solid and liquid gain media in our integrated ring-waveguide platform. This micro-ring resonator is spectrally agile, allowing changes in the lasing wavelength through a simple procedure, offering an attractive feature for complex on-chip photonic networks. As further demonstrations of this we investigated the emission spectra of a resonator filled with three mixtures, namely CY3-doped solid SU-8, CY3-doped liquid SU-8, and CY3-doped toluene. Figure 7 displays the multimode laser output spectra centered around 550 nm and observed at various pump intensities, together with the spectrally integrated laser emission recorded at the output port of the waveguide using an Ocean Optics HR4000 spectrometer (Spectrometer 1 in Fig. 4). The extracted lasing thresholds for CY3-doped solid SU-8, CY3-doped liquid SU-8, and CY3-doped toluene ring lasers were approximately 630 nJ/mm^2^, 1.25 μJ/mm^2^, and 2.5 μJ/mm^2^, respectively.

## Discussion

The integration of reusable and wavelength-agile ring resonator lasers with laser-inscribed photonic waveguides has been successfully demonstrated. The solid-state ring lasers offer competitive benefits when compared to other integrated solid-state dye lasers, such as widely-tunable emission from a single chip, low lasing thresholds, inherent mechanical robustness, and a platform that can be used repeatedly. Our unique design also accommodates fluidic phase gain media, making it compatible with future applications in the blossoming field of optofluidics. Further studies will seek to incorporate a second liquid ring resonator nearby to achieve single-mode of laser operation via the Vernier effect^1^,^2^,^10^ and implement a fully integrated 3-D microfluidic system as presented in our recent work^9^. A real-time wavelength-tunable optofluidic laser will be achieved by altering the solution in one of the rings. All advanced features of this laser platform are complemented with the uniquely inscribed photonic waveguides that can be distributed in the entire volume of the substrate with unparalleled spatial density^27^,^28^.

The successful implementation of ultrafast laser inscribed photonic waveguides to direct laser output will enable future on-chip optical signal processing with much higher complexity. The physical isolation of waveguides from the laser resonator and gain volume allows convenient, economic, rapid and accurate patterning with a one-step, maskless ultrafast laser writing process at room temperature. This approach will facilitate enhanced intra- and inter-chip optical connectivity. Furthermore, more sophisticated structures on ring resonators such as gratings can be explored^29^. Finally, this level of integration promises to facilitate the investigation at low input powers of novel nonlinear phenomena, such as parity-time (*PT*) symmetry in optics^30^,^31^, light induced transparency^32^,^33^, and molecular magneto-electric effects^34^,^35^,^36^ on photonic chips.

## Additional Information

**How to cite this article**: Chandrahalim, H. *et al*. Fusion of Renewable Ring Resonator Lasers and Ultrafast Laser Inscribed Photonic Waveguides. *Sci. Rep.* **6**, 32668; doi: 10.1038/srep32668 (2016).

## Supplementary Material

Supplementary Information

## References

[b1] Lee W. . Tunable single mode lasing from an on-chip optofluidic ring resonator laser. Appl. Phys. Lett. 98, 061103 (2011).

[b2] Li Z. In Frequency Control Symposium (FCS), IEEE International. 1–4 (2012).

[b3] Kuwata-Gonokami M. . Polymer microdisk and microring lasers. Opt. Lett. 20, 2093–2095 (1995). 19862261 10.1364/ol.20.002093

[b4] Lu S.-Y. . Highly stable on-chip embedded organic whispering gallery mode lasers. J. Lightwave Technol. 32, 2415–2419 (2014).

[b5] Ku J.-F. . Photonic-molecule single-mode laser. IEEE Photon. Technol. Lett. 27, 1157–1160 (2015).

[b6] Bog U. . Large‐Scale Parallel Surface Functionalization of Goblet‐type Whispering Gallery Mode Microcavity Arrays for Biosensing Applications. Small 10, 3863–3868 (2014). 24990526 10.1002/smll.201400813

[b7] Bog U. . Densely Packed Microgoblet Laser Pairs for Cross‐Referenced Biomolecular Detection. Adv. Sci. 2, 1500066 (2015). 10.1002/advs.201500066 PMC5033028 27708994

[b8] Wienhold T. . All-polymer photonic sensing platform based on whispering-gallery mode microgoblet lasers. Lab Chip 15, 3800–3806 (2015). 26266577 10.1039/c5lc00670h

[b9] Chandrahalim H., Chen Q., Said A. A., Dugan M. & Fan X. Monolithic optofluidic ring resonator lasers created by femtosecond laser nanofabrication. Lab Chip 15, 2335–2340 (2015). 25904381 10.1039/c5lc00254k PMC4422773

[b10] Chandrahalim H. & Fan X. Reconfigurable solid-state dye-doped polymer ring resonator lasers. Sci. Rep. 5, 18310 (2015). 26674508 10.1038/srep18310 PMC4682137

[b11] Hausmann B. J. . Integrated high-quality factor optical resonators in diamond. Nano Lett. 13, 1898–1902 (2013). 23427820 10.1021/nl3037454

[b12] Hosseini E. S., Yegnanarayanan S., Atabaki A. H., Soltani M. & Adibi A. High quality planar silicon nitride microdisk resonators for integrated photonics in the visible wavelength range. Opt. Express 17, 14543–14551 (2009). 19687933 10.1364/oe.17.014543

[b13] Kiyat I., Aydinli A. & Dagli N. High-Q silicon-on-insulator optical rib waveguide racetrack resonators. Opt. Express 13, 1900–1905 (2005). 19495071 10.1364/opex.13.001900

[b14] Webster M., Pafchek R., Sukumaran G. & Koch T. Low-loss quasi-planar ridge waveguides formed on thin silicon-on-insulator. Appl. Phys. Lett. 87, 231108 (2005).

[b15] Testa G., Collini C., Lorenzelli L. & Bernini R. Planar Silicon-Polydimethylsiloxane Optofluidic Ring Resonator Sensors. IEEE Photon. Technol. Lett. 28, 155–158 (2016).

[b16] Testa G., Huang Y., Sarro P. M., Zeni L. & Bernini R. Integrated silicon optofluidic ring resonator. Appl. Phys. Lett. 97, 131110 (2010).

[b17] Testa G., Persichetti G. & Bernini R. Optofluidic approaches for enhanced microsensor performances. Sensors 15, 465–484 (2014). 25558989 10.3390/s150100465 PMC4327030

[b18] Absil P. . Vertically coupled microring resonators using polymer wafer bonding. IEEE Photon. Technol. Lett. 13, 49–51 (2001).

[b19] Little B. . Very high-order microring resonator filters for WDM applications. IEEE Photon. Technol. Lett. 16, 2263–2265 (2004).

[b20] Suzuki S., Shuto K. & Hibino Y. Integrated-optic ring resonators with two stacked layers of silica waveguide on Si. IEEE Photon. Technol. Lett. 4, 1256–1258 (1992).

[b21] Tishinin D. . Vertical resonant couplers with precise coupling efficiency control fabricated by wafer bonding. IEEE Photon. Technol. Lett. 11, 1003–1005 (1999).

[b22] Yanagase Y., Suzuki S., Kokubun Y. & Chu S. T. Box-like filter response and expansion of FSR by a vertically triple coupled microring resonator filter. J. Lightwave Technol. 20, 1525–1529 (2002).

[b23] Kokubun Y., Hatakeyama Y., Ogata M., Suzuki S. & Zaize N. Fabrication technologies for vertically coupled microring resonator with multilevel crossing busline and ultracompact-ring radius. IEEE J. Sel. Top. Quantum Electron. 11, 4–10 (2005).

[b24] Bellouard Y., Said A. & Bado P. Integrating optics and micro-mechanics in a single substrate: a step toward monolithic integration in fused silica. Opt. Express 13, 6635–6644 (2005). 19498678 10.1364/opex.13.006635

[b25] Bado P., Said A. & Dugan M. In International Congress on Applications of Lasers & Electro-Optics (ICALEO). 1–9 (2003).

[b26] Bado P., Said A., Dugan M., Sosnowski T. & Wright S. In The National Fiber Optic Engineering Conference (NFOEC). 1153–1158 (2002).

[b27] Thomson R., Birks T. A., Leon-Saval S., Kar A. & Bland-Hawthorn J. Ultrafast laser inscription of an integrated photonic lantern. Opt. Express 19, 5698–5705 (2011). 21445210 10.1364/OE.19.005698

[b28] Thomson R. . Ultrafast laser inscription of a 121-waveguide fan-out for astrophotonics. Opt. Lett. 37, 2331–2333 (2012). 22739898 10.1364/OL.37.002331

[b29] Arbabi A., Kamali S. M., Arbabi E., Griffin B. G. & Goddard L. L. Grating integrated single mode microring laser. Opt. Express 23, 5335–5347 (2015). 25836565 10.1364/OE.23.005335

[b30] Peng B. . Loss-induced suppression and revival of lasing. Science 346, 328–332 (2014). 25324384 10.1126/science.1258004

[b31] Peng B. . Parity–time-symmetric whispering-gallery microcavities. Nat. Phys. 10, 394–398 (2014).

[b32] Kim J., Kuzyk M. C., Han K., Wang H. & Bahl G. Non-reciprocal Brillouin scattering induced transparency. Nat. Phys. 11, 275–280 (2015).

[b33] Peng B., Özdemir Ş. K., Chen W., Nori F. & Yang L. What is and what is not electromagnetically induced transparency in whispering-gallery microcavities. Nat. Commun. 5, 5082 (2014). 25342088 10.1038/ncomms6082

[b34] Rand S. C., Fisher W. & Oliveira S. L. Optically induced magnetization in homogeneous, undoped dielectric media. J. Opt. Soc. Am. B 25, 1106–1117 (2008).

[b35] Chakrabarty A. . In Frontiers in Optics. FW3E. 5 (Optical Society of America).

[b36] Fisher A., Cloos E., Fisher W. & Rand S. Dynamic symmetry-breaking in a simple quantum model of magneto-electric rectification, optical magnetization, and harmonic generation. Opt. Express 22, 2910–2924 (2014). 24663583 10.1364/OE.22.002910

