# Supplementary material for: Fusion of Renewable Ring Resonator Lasers and Ultrafast Laser Inscribed Photonic Waveguides
Source: Sci Rep. 2016 Sep 7;6:32668. doi: 10.1038/srep32668 (PMC5013411; doi:10.1038/srep32668)
Supplement: Supplementary Information [file srep32668-s1.pdf]

# Supplementary Information

# Fusion of Renewable Ring Resonator Lasers and Ultrafast Laser Inscribed Photonic Waveguides

Hengky Chandrahalim[1,2], Stephen C. Rand[2], and Xudong Fan[1*]

[1]Department of Biomedical Engineering, University of Michigan,

1101 Beal Ave., Ann Arbor, MI 48109, USA

[2]Department of Electrical Engineering and Computer Science, University of Michigan,

1301 Beal Ave., Ann Arbor, MI 48109, USA

*xsfan@umich.edu

We estimated the coupling efficiency of our waveguide to the polymer ring resonator according to the formula[1]:

$$\kappa^2 = \left[\frac{2\pi R}{\gamma} e^{\left(-\frac{(\Delta\beta)^2 R}{\gamma}\right)}\right] \frac{k^4}{4\beta_R \beta_{WG}} ({n_{WG}}^2 - {n_{FS}}^2)({n_R}^2 - {n_{FS}}^2) \int_{WG} e_{WG}\, e_R\, dx \int_R \, e_{WG}\, e_R\, dx \qquad \text{(S1)}$$

The subscripts R, WG, and FS refer to the ring resonator, waveguide, and the fused-silica substrate, respectively. The evanescently decayed electric field distributions in the waveguide and ring resonator are described by $e_{WG}$ and $e_R$, respectively, where both fields are normalized to their respective total intensity in the x-y plane. All parameters that we use to estimate the coupling efficiency between the waveguide and the ring resonator are tabulated in Table S1.

**Table S1.** Parameters used to analyze the coupling efficiency between the optical waveguide and polymer ring resonator

| Parameter | Definition | Value | Unit |
|---|---|---|---|
| $\lambda$ | Wavelength of operation | 560 | nm |
| R | Ring radius | 150 | μm |
| $\gamma$ | Decay constant of RR | 7.86 | $\mu m^{-1}$ |
| $\beta_R$ | Propagation constant of the RR | 16.6 | $\mu m^{-1}$ |
| $\beta_{WG}$ | Propagation constant of the WG | 16.4 | $\mu m^{-1}$ |
| $\Delta\beta$ | $\beta_R$ - $\beta_{WG}$ | 0.2 | $\mu m^{-1}$ |
| k | $2\pi/\lambda$ | 11.2 | $\mu m^{-1}$ |
| $n_R$ | Refractive index of RR | 1.62 | – |
| $n_{WG}$ | Refractive index of WG | 1.47 | – |
| $n_{FS}$ | Refractive index of FS | 1.46 | – |
| $e_R$ | Guided mode decay in RR | – | – |
| $e_{WG}$ | Guided mode decay in WG | – | – |
| $\kappa$ | Coupling coefficient | Tabulated in Table S2 | – |

We analytically modeled the evanescently decayed fields in the ring resonator and waveguide and calculated the overlap integral for different coupling gaps as presented in Fig. S1.

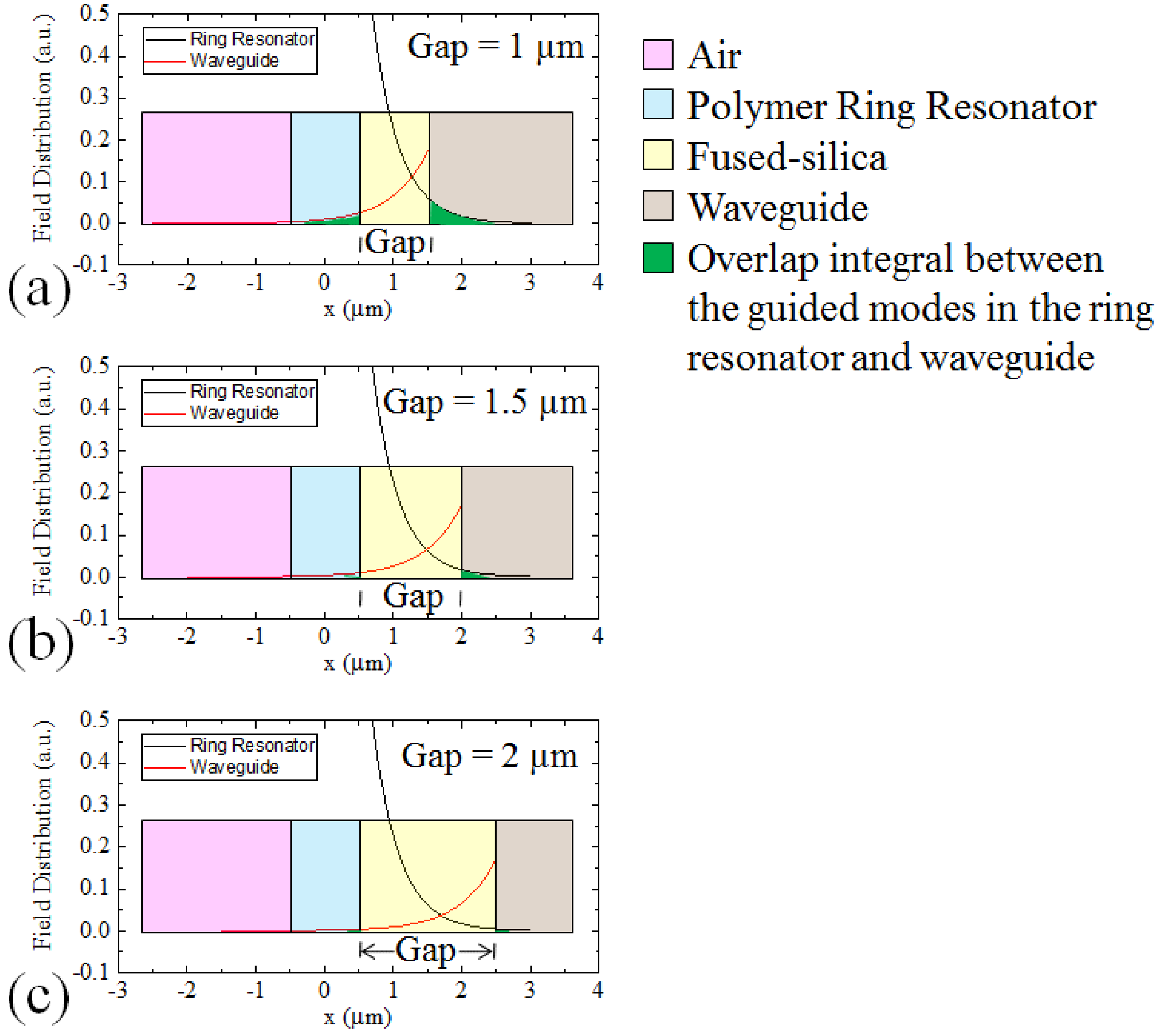


**Fig. S1.** The overlap integral of the evanescently decayed fields in the ring resonator and waveguide for (a) 1 µm, (b) 1.5 µm, and (c) 2 µm coupling gap.

We estimated the coupling coefficient κ as a function of various coupling gaps and plotted the results in Fig. S2.

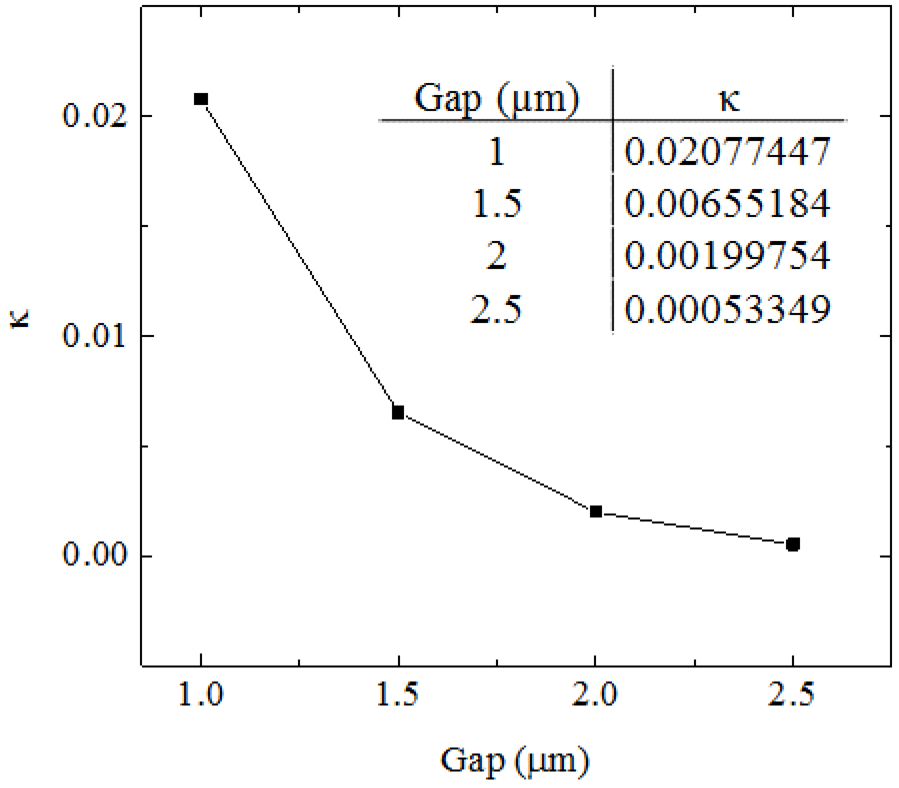


**Fig. S2.** Coupling coefficient between the ring resonator and optical waveguide as a function of the coupling gap.

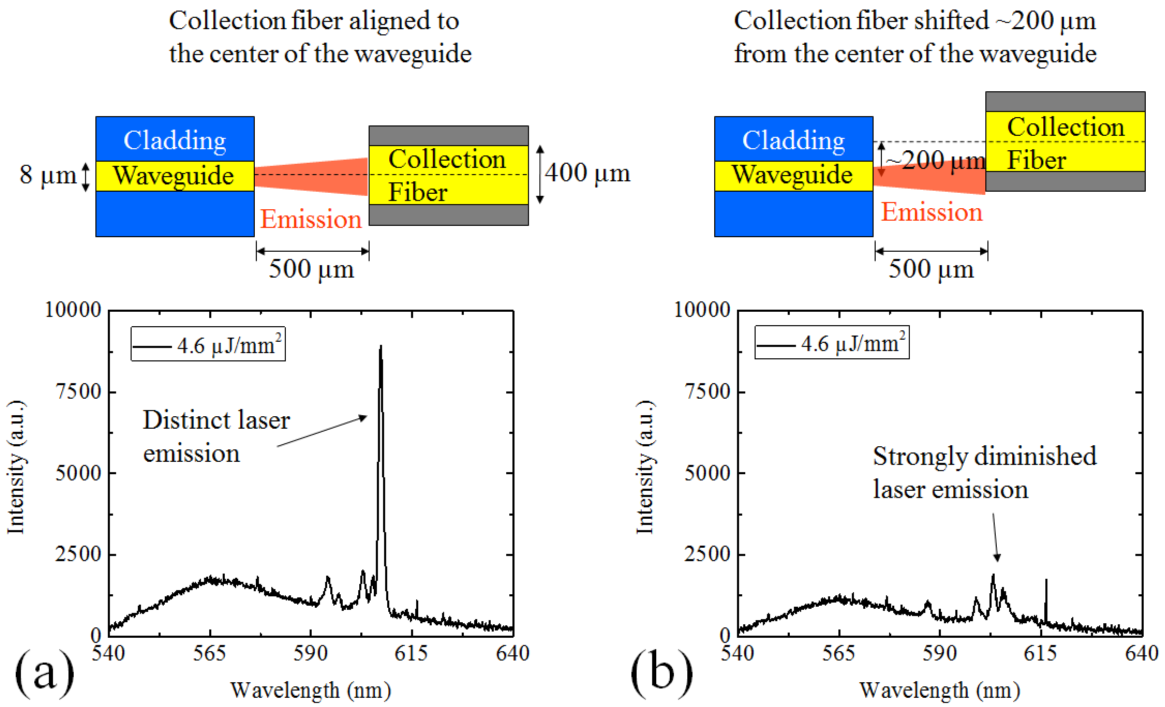


**Fig. S3.** (a) The guided lasing spectrum of a solid R6G-doped SU-8 ring laser when the multimode collection fiber was aligned to the center of the refractive index modified waveguide. (b) The guided lasing spectrum of the same ring laser in (a) when the multimode collection fiber was shifted approximately 200 μm away from the center of the waveguide. The distance between the collection fiber and the waveguide was maintained at ~500 μm for both experiments. The lasing spectrum was detected at the output port of the optical waveguide by Ocean Optics HR4000 spectrometer with 0.7 nm resolution (Spectrometer 1 in Fig. 4). The pump intensity of 4.6 μJ/mm$^2$ at 530 nm wavelength was applied to both measurements in (a) and (b).

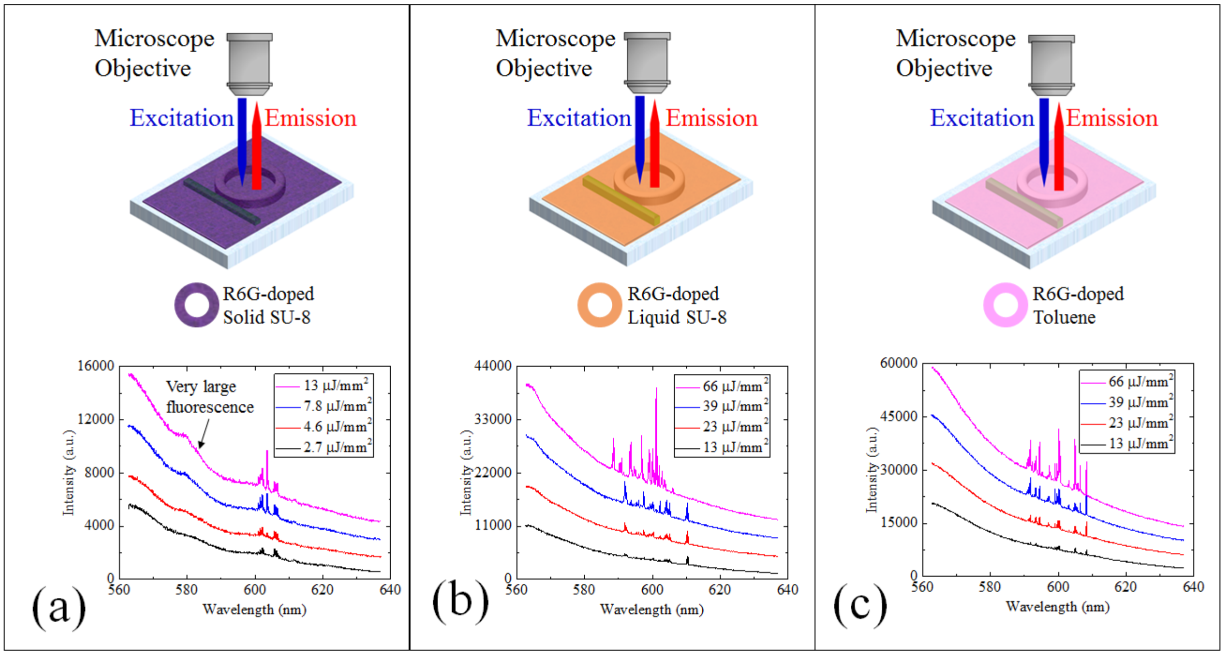


**Fig. S4.** Free-space detected lasing spectra of the same three ring cavity lasers presented in Fig. 6. The unguided lasing spectra of R6G-doped solid SU-8 (a), R6G-doped liquid SU-8 (b), and R6G-doped toluene (c) ring resonator lasers were detected from the top of the device with a microscope objective. A Horiba iHR550 spectrometer (Spectrometer 2 in Fig. 4) with a 600 g/mm grating was used to record the multimode laser emissions along with fluorescent background from the dye molecules. The spot size of the OPO pumping laser beam was approximately 0.03 $mm^2$. All spectra are vertically shifted for clarity. The excitation wavelength for all measurements in (a-c) was 530 nm.

## References


1 White, I. M., Oveys, H., Fan, X., Smith, T. L. & Zhang, J. Integrated multiplexed biosensors based on liquid core optical ring resonators and antiresonant reflecting optical waveguides. *Appl. Phys. Lett.* **89**, 191106 (2006).
